# Exploring the Diagnostic Spectrum of Children with Raised Faecal Calprotectin Levels

**DOI:** 10.3390/children11040420

**Published:** 2024-04-02

**Authors:** Angharad Vernon-Roberts, Olivia Humphrey, Andrew S. Day

**Affiliations:** 1 Department of Paediatrics, University of Otago Christchurch, Christchurch 8011, New Zealand; angharad.hurley@otago.ac.nz; 2 Christchurch Hospital, Te Whatu Ora Waitaha Canterbury, Christchurch 8011, New Zealand

**Keywords:** children, calprotectin, alarm symptoms, diagnostic

## Abstract

Faecal calprotectin (FC) is a marker of gut inflammation. The cause and relevance of raised FC in children outside the context of established inflammatory bowel disease (IBD) have had minimal attention. This study aimed to address this by carrying out a retrospective study on children with abnormal FC tests aged 4–17 years without established IBD in the South Island, New Zealand. Abnormal FC results were stratified: 51–249 μg/g, 250–499 μg/g, and 500+ μg/g, and participants were categorised into diagnostic groups. Data were collected on symptoms and diagnostic tests. Three-hundred and ten children had abnormal index FC results, with a mean age of 12.9 years, and a 55% proportion of females. The median FC was 125 μg/g; 71% had levels 51–249 μg/g and 21% had levels 500+ μg/g. Of those with FC 500+ μg/g, 89% either had infectious diarrhoea or were diagnosed with IBD at the time of, or subsequent to, the index FC. Alarm symptoms did not delineate between groups with FC 500+ μg/g. Abnormalities in platelet levels, abdominal ultrasound, and colonoscopy were more frequent for children diagnosed with IBD. Repeat FC test levels were significantly reduced except for those subsequently diagnosed with IBD. Abnormal FC levels for the majority were below the level indicative of mucosal inflammation. Repeat FC testing could play an important role in distinguishing between diagnoses.

## 1. Introduction

Children experiencing chronic or acute gastrointestinal (GI) symptoms are a frequent cause of presentation to primary or emergency care and ensuing referrals to tertiary care specialists [1]. Testing strategies to differentiate between inflammatory, infectious, or functional causes of GI symptoms vary greatly, and there is little evidence on how abnormal tests such as stool and serum inflammatory markers may correlate with radiological testing, endoscopic investigation, or presenting symptoms, to aid clinicians in differentiating between diagnoses.

One laboratory marker known to correlate well with inflammation levels in the GI tract is calprotectin, a zinc- and calcium-binding protein from the S100 family that is mainly present in neutrophils and is released during the process of inflammation [2,3]. When measured in stool, faecal calprotectin (FC) is considered a highly reliable non-invasive biomarker of GI inflammation as calprotectin is released from neutrophils that have migrated into the bowel lumen [3]. FC varies in children as it is typically elevated at birth and then decreases until stabilising at approximately four years of age [4,5]. The FC level considered ‘normal’ after the age of four years is ≤50 μg/g, with levels >250 μg/g being indicative of intestinal inflammation [6]. FC levels may be raised in children due to a number of conditions, such as inflammatory bowel disease (IBD), infectious diarrhoea, necrotising enterocolitis, human immunodeficiency virus, the use of non-steroidal anti-inflammatory drugs, and intestinal inflammation in other conditions such as cystic fibrosis [7,8,9].

FC within the context of paediatric IBD is widely researched, and the evidence base is building for the utility of FC in differentiating between inflammatory conditions and functional gastrointestinal disorders (FGID) [10,11]. However, the correlation between FC at different levels and the components of the diagnostic process for children presenting with GI symptoms is infrequently studied among children without IBD. The aim of this study was to determine associations between elevated FC levels, symptoms, inflammatory markers, endoscopy, and radiological investigations that are carried out as part of the diagnostic process in a cohort of children without a pre-existing diagnosis of IBD in New Zealand.

## 2. Materials and Methods

### 2.1. Study Centre and Data Acquisition

The study was carried out at Christchurch Hospital, Te Whatu Ora, Waitaha Canterbury, New Zealand. Data on all FC tests carried out on children and analysed at the Canterbury Health Laboratories (CHL) between January 2018 and July 2020 were requested and provided. FC tests were considered ‘index’ if they were the initial FC test carried out, and a ‘repeat’ if a follow-up test had been carried out within 180 days of the index FC test. Those repeated outside this parameter were excluded due to a reduction in clinical significance after six months.

### 2.2. Study Population

FC test results were included in the data analysis if they satisfied the following criteria.

#### 2.2.1. Inclusion Criteria

Children were included if they had an index FC level >50 μg/g and were aged between four and seventeen years of age at the time of the test.

#### 2.2.2. Exclusion Criteria

Children were excluded from the study if they had an established diagnosis of IBD, and were aged less than four years, or eighteen years and over, at the time of the test. Children were excluded if they resided in the North Island of New Zealand while their testing was carried out in CHL (South Island), as hospital records are region-specific and not available to clinicians or researchers in the South Island.

### 2.3. Data Collected

Diagnostic and testing data were collected for each study participant using the Christchurch Hospital electronic medical record (EMR). Data included the following:

- Demographic information.
- Presenting symptoms.
- Serum tests carried out within one week of the index FC test were recorded as abnormal if outside the stated laboratory reference range.
  – Haemoglobin (Hb)
  – White cell count (WCC)
  – Platelets
  – C-reactive protein (CRP)
  – Erythrocyte sedimentation rate (ESR)
  – Albumin
  – Iron studies.
- Radiological investigations carried out within two weeks of the index FC test were recorded as abnormal if there were findings recorded outside the normal expected parameters.
- Endoscopic investigations carried out within four weeks of the index FC test were recorded as abnormal if there were findings recorded outside the normal expected parameters. The findings for gastroscopies and colonoscopies were recorded separately even if the procedure included both upper and lower endoscopies.

### 2.4. Diagnosis Based on Index FC Test

Longitudinal follow-up for 18 months after index FC was conducted to confirm if the initial diagnosis changed.

### 2.5. Outcomes

#### 2.5.1. FC Levels

FC results were categorised into three groups for analysis:

- A range of 51–249 μg/g to represent the lower levels of FC and to account for the proposed threshold of an IBD diagnosis being >212 μg/g [12]
- A range of 250–499 μg/to account for levels >250 μg/g considered predictive of mucosal inflammation
- All values ≥500 μg/g, as the Canterbury Health Laboratory categorised FC levels as ‘very high’ at different levels throughout the study period (500+, 800+, 1000+), so all results ≥500 μg/g were classified together.

#### 2.5.2. Diagnostic Groups

All participants were assigned to one of seven diagnostic groups based on their immediate records at the time of index FC, and this was confirmed with longitudinal follow-up from hospital EMR up to 18 months after index FC test. The groups were assigned as follows:

- Functional if the diagnosis was aligned with Rome IV criteria categories [13].
- Infectious if the diagnosis was stated as ‘viral’ gastroenteritis with no diagnostic stool tests carried out, or if it was confirmed on additional stool tests as being parasitic, viral, or bacterial in origin.
- Upper/lower GI if diagnosed with an acute or chronic GI condition other than IBD.
- Non-GI if diagnosed with a condition not related to the GI tract.
- Unknown if the cause was not found, or the child was managed by the primary care physician and primary care records were not available.
- IBD diagnostic if the index FC was specifically related to an IBD diagnosis within 8 weeks of the index FC test.
- IBD subsequent if the participant received an IBD diagnosis during longitudinal follow-up after eight weeks from index FC.

#### 2.5.3. Symptoms

Presenting Symptoms

Data on symptoms were categorised by location into upper GI, lower GI, general GI, or extra-intestinal manifestations (EIM).

Alarm Symptoms

Symptoms specifically considered ‘alarm’ symptoms that are proposed to indicate an increased likelihood of IBD [14], and other organic conditions [13], were also presented: rectal bleeding/bleeding diarrhoea (classified as PR bleeding), abdominal pain, weight loss, and diarrhoea.

#### 2.5.4. Additional Tests

Where additional laboratory, radiological, and endoscopic investigations were carried out and found to be abnormal (over/under the laboratory reference value, or abnormal findings), these were studied for their association with the FC level groups.

### 2.6. Ethics

Ethics approval for the study was provided by the University of Otago Human Ethics Committee (Health), Dunedin, New Zealand [HD18/084].

### 2.7. Statistical Analysis

Basic demographic and descriptive data are presented as a mean (standard deviation (SD)) or number and percentage of the cohort or an appropriate denominator (N (%) or n/N (%)). FC levels are presented as a median (inter-quartile range as 25th–75th percentile) as FC levels are known to be highly skewed. FC group categorical comparisons were made using contingency tables with a Chi (χ^2^) or Pearson test and the associated effect size (Phi), with results *p* < 0.05 considered significant. Statements of assumption on the direction of significance were reported based on the observed and expected cell counts in contingency tables. Data analyses were carried out using SPSS version 29.0 (IBM Corp.; Armonk, NY, USA) and graphs developed using Microsoft Excel 365 Enterprise and SPSS version 29.0 (IBM Corp.; Armonk, NY, USA).

## 3. Results

### 3.1. Faecal Calprotectin Tests

Laboratory data were provided for 2475 FC tests for children between January 2018 and July 2020 inclusive. From these tests, 1428 were either ≤50 μg/g or had an insufficient sample, and 1047 were >50 μg/g. Of the 1047, 628 tests were excluded during the review process, of which 275 were follow-up tests for children with an established IBD diagnosis (Figure S1). A total of 398 valid tests were available for inclusion in the data analysis, and of these, 310 were index tests (initial FC test carried out) for individual participants and 88 were included as repeat tests within the stated time frame of 180 days.

### 3.2. Study Population

The mean age of the 310 children with a positive FC index test (>50 μg/g) was 12.9 years (SD 4.2), and 170 (55%) were female. Fifty-one (17%) had a known chronic condition, and all ethnicity groups were represented: NZ European, 272 (87%); Māori, 31 (10%); Pacifica, 5 (0.2%); Asian, 18 (0.6%); Middle Eastern, Latin American, and African, 6 (0.2%); and Other, 35 (11%).

### 3.3. Diagnostic Grouping

For the overall cohort, the diagnosis associated with each individual’s index FC test was extracted from hospital records and allocated to one of seven categories. The distribution in these groups was 57 (18%) with a stated functional GI disorder; 33 (11%) had a stated or proven GI infection; 48 (15%) had an upper/lower GI condition (not IBD); 20 (6%) had a non-GI condition; 83 (27%) were categorised as unknown due to having no confirmed diagnosis or no hospital level records available; for 53 (17%), the FC was part of the diagnostic work-up for IBD that was confirmed within 8 weeks of the index FC; and 16 (5%) were diagnosed with subsequent IBD between 2–15 months after the index test (Table 1).

### 3.4. Index FC Test Levels for Overall Cohort

**Figure 1:**
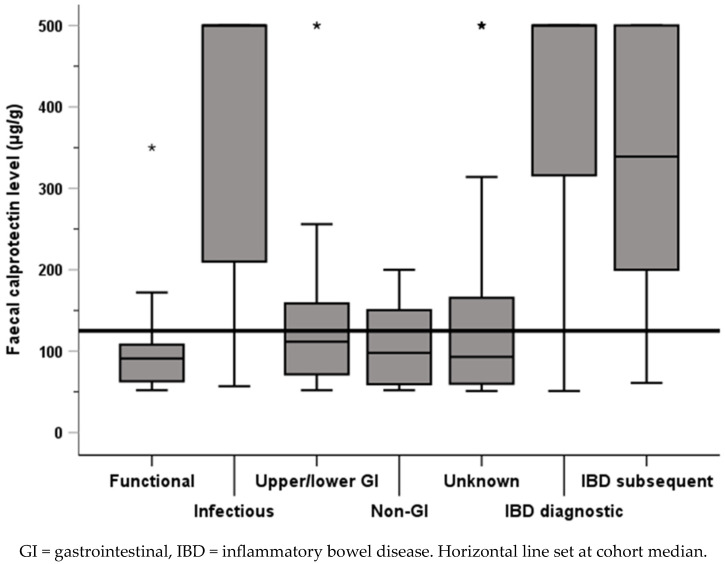
FC levels by diagnostic group, with comparison against cohort median.

The median index FC level (minimum 51, maximum 500) was 125 μg/g (IQR 71 to 348). Three groups (Infectious, IBD diagnostic, and IBD subsequent) had higher median levels than seen in the overall cohort, while the remaining groups (functional, upper/lower GI, non-GI, and unknown) had lower median levels (all <115 μg/g) (Figure 1).

**Figure 2:**
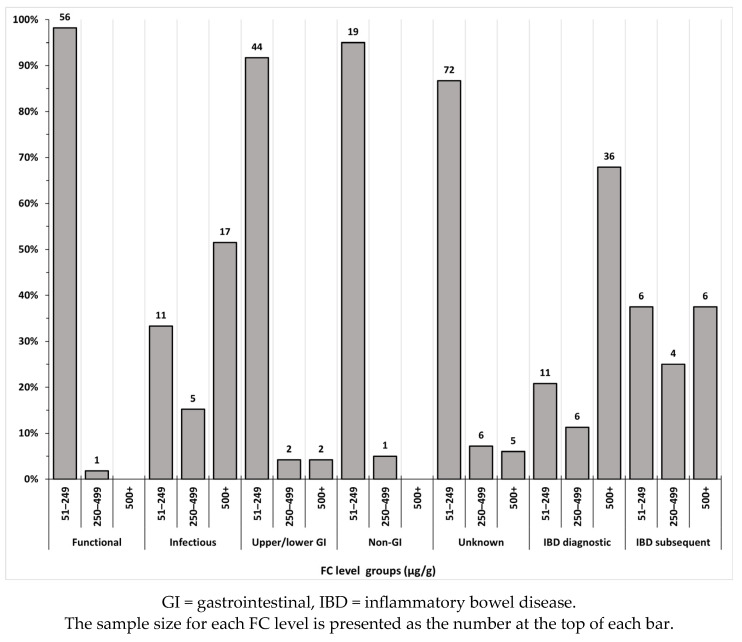
FC level cut-offs for each of the seven diagnostic groups among the study cohort.

When the FC levels were categorised into groups, the majority of the cohort (219 (71%)) had FC levels between 51 and 249 μg/g, 25 (8%) between 250 and 499 μg/g, and 66 (21%) at levels 500+ μg/g. In the diagnostic groups of functional GI, GI upper/lower, non-GI, and unknown, over 85% of group participants had FC levels <250 μg/g; FC levels for children in the infectious, IBD diagnostic, and IBD subsequent groups were ≥250 μg/g for at least 60% of children in each and accounted for 89% of levels 500+ μg/g (Figure 2). 

### 3.5. Symptoms

#### 3.5.1. Presenting Symptoms

Diarrhoea and abdominal pain were the most frequently observed presenting symptoms for the whole cohort (Table 2). Symptoms were categorised as lower GI symptoms, upper GI symptoms, general GI symptoms, and extra-intestinal symptoms with comparisons made between FC level groups. 

**Figure 3:**
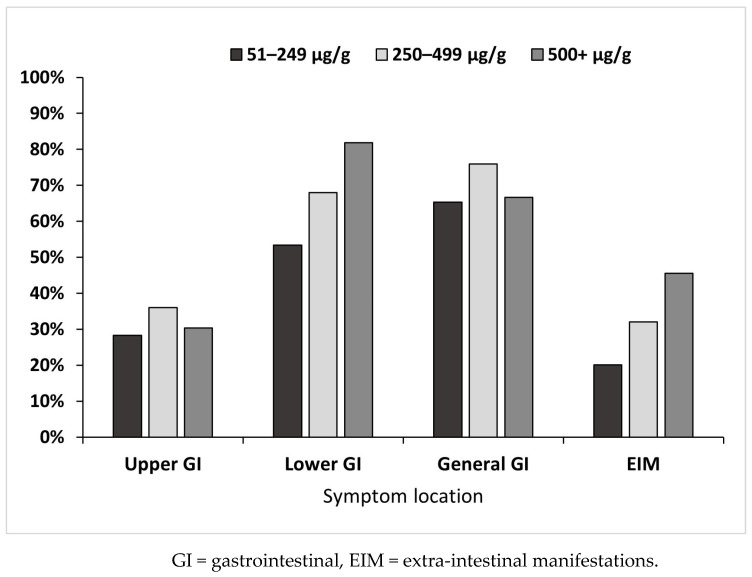
The percentage of children experiencing symptom categories in each FC level group, expressed as a binary variable (experienced/not experienced).

There was no association between the number of children experiencing upper GI symptoms between the FC level groups (χ^2^ 0.68, Phi 0.05, *p* = 0.71) or general GI symptoms (χ^2^ 1.16, Phi 0.06, *p* = 0.56) (Figure 3). However, children with FC levels ≥500 μg/g were more likely to experience lower GI symptoms (χ^2^ 17.75, Phi 0.24, *p* < 0.001) and EIM (χ^2^ 17.2, Phi 0.24, *p* < 0.001) than the other FC level groups (Figure 3). 

#### 3.5.2. Alarm Symptoms

Alarm symptoms experienced according to FC level groups

**Figure 4:**
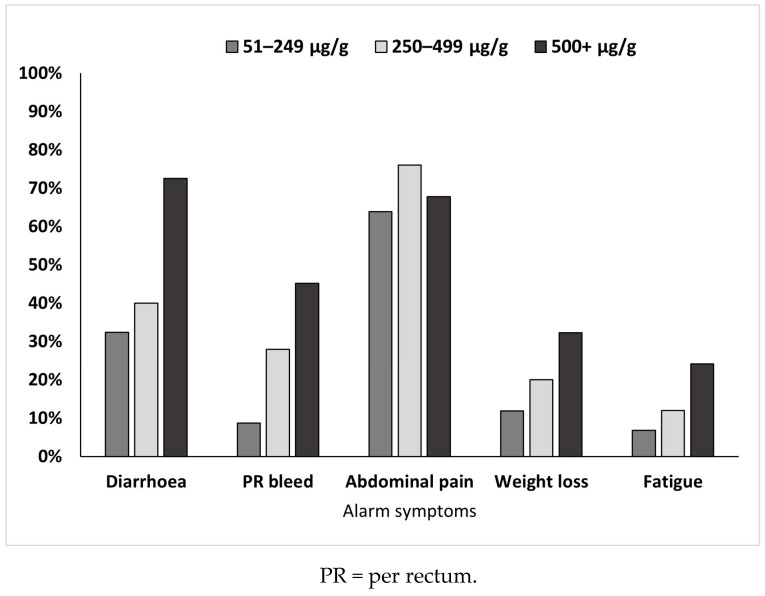
The percentage of children in each FC level group experiencing ‘alarm’ symptoms.

When the symptoms were looked at specifically through the lens of what are considered ‘alarm symptoms’ and compared between the different FC levels, the percentage of children experiencing abdominal pain did not differ between the groups (χ^2^ 1.49, Phi 0.07, *p* = 0.48) (Figure 4). However, more children in the FC 500+ μg/g group experienced diarrhoea (χ^2^ 26.89, Phi 0.26, *p* < 0.001), PR bleed (χ^2^ 42.27, Phi 0.37, *p* < 0.001), weight loss (χ^2^ 12.78, Phi 0.20, *p* = 0.002), and fatigue (χ^2^ 13.49, Phi 0.21, *p* = 0.001) than in the other FC level groups.

Number of alarm symptoms experienced

**Figure 5:**
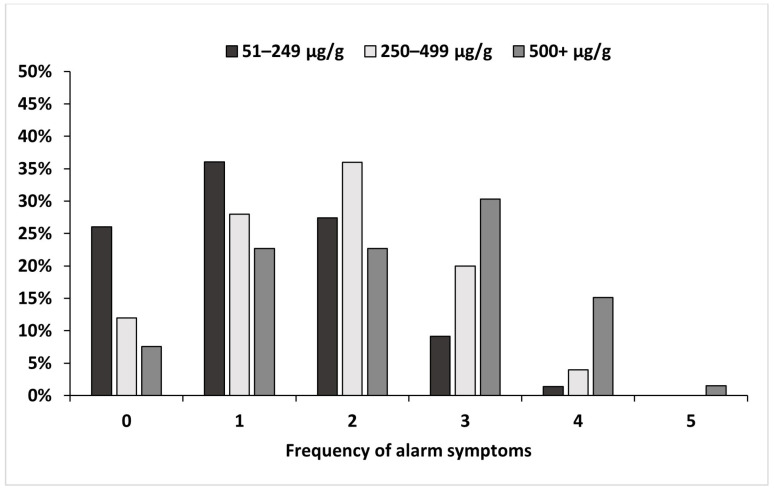
Frequency of alarm symptoms overall for each FC group.

The number of alarm symptoms experienced by children in each FC and diagnostic group varied (Figure S2a,b), with all FC level groups having children experiencing up to four alarm symptoms, with a greater frequency seen in the 500+ μg/g group (Figure 5).

Alarm symptoms experienced according to diagnostic groups

Alarm symptoms were experienced by at least 25% of children in the IBD diagnostic and IBD subsequent groups but less consistently in the other groups (Figure S3). However, when sub-group comparisons were made between the infectious, IBD diagnostic, and IBD subsequent groups, there was no association between the three groups for any of the individual alarm symptoms (all *p* > 0.1). 

### 3.6. Additional Tests Carried out on Overall Cohort

The additional tests carried out on the study cohort were examined, with the percentage performed and proportion that were abnormal/normal in each FC level group presented (Figure S4). This was also examined at the individual and diagnostic group levels (Figure S5a,b). Most serum tests were abnormal for more children in the FC group 500+ μg/g compared to both of the other groups (Table 3). One-hundred and two (33%) children had radiological testing as part of their diagnostic process (Figure S4). Endoscopies were carried out on 129 children, with 17 (5%) having gastroscopies, 32 (10%) having colonoscopies, and 80 (26%) having both procedures (overall: 97 gastroscopies and 112 colonoscopies). More children in the 500+ μg/g group had abnormal magnetic resonance imaging (MRI) of the abdomen/small bowel and abdominal USSs, and more abnormal colonoscopy findings, than in the other groups (Table 3). 

Within the infectious GI (N = 33), IBD diagnostic (N = 53), and IBD subsequent (N = 16) diagnostic groups, additional analysis was carried out to help differentiate between them, as these groups represented the highest frequency in the 500+ μg/g FC level group. This showed that the proportion of abnormal tests (of those that had each test conducted) between groups differed only for platelets (χ^2^ 10.24, Phi 0.38, *p* = 0.006), abdominal USSs (χ^2^ 10.83, Phi 0.63, *p* = 0.004), and colonoscopy (χ^2^ 15.97, Phi 0.50, *p* ≤ 0.001), with the IBD diagnostic group having more abnormal findings for all three investigations than the other two groups (Figure S5b).

### 3.7. Retest FC for Sub-Group of the Cohort 

**Figure 6:**
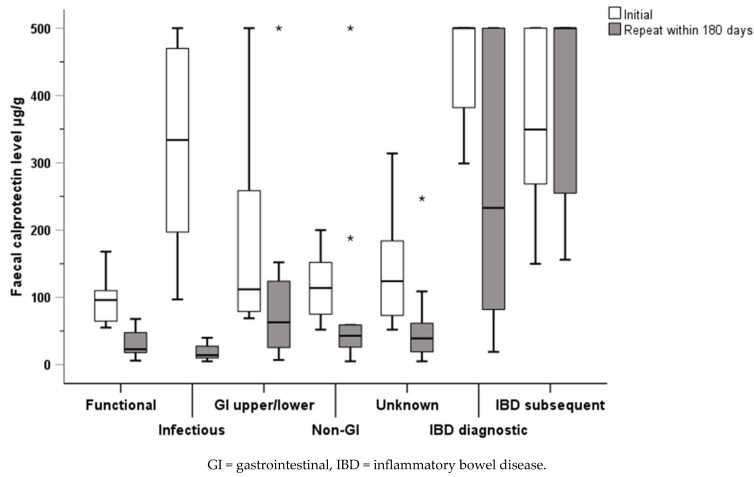
Index and repeat FC retest levels within 180 days across diagnostic groups.

Repeat FC testing was carried out on 109 participants, but only 88 (28% of the overall cohort) were within the required 180-day study period and included in the analysis. There was a median of 68.5 days (IQR 32.3 to 106.8) between tests and 53 (60%) were tested within 60 days of the index FC. Participants from all diagnostic groups had repeat FC testing: functional, 9 (10%); infectious, 9 (10%); upper/lower GI, 8 (9%); non-GI, 7 (8%); unknown, 21 (24%); IBD diagnostic (repeats now post-treatment), 22 (25%); and IBD subsequent, 12 (14%). With the exception of the IBD subsequent group, all group FC medians were reduced on repeat testing (Figure 6). 

**Figure 7:**
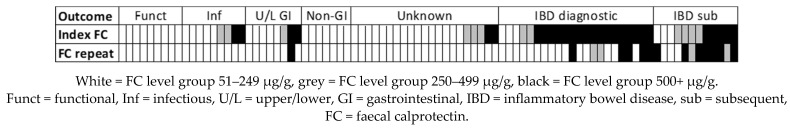
Individual repeat testing results for children in each diagnostic group.

When individual data for FC level groups were examined, it could be seen that participants in the functional, infectious, and unknown groups all had repeat tests in the FC group 51–249 μg/g regardless of the FC group of their index FC. One participant in the upper/lower GI group had an index and repeat FC level in the 500+ μg/g group: this child was diagnosed with NSAID enteropathy with repeat testing 136 days after the index test. There was a more complex pattern of repeat testing results in the two IBD groups (Figure 7).

## 4. Discussion

This retrospective evaluation of the outcomes of FC testing carried out as part of the diagnostic process for a large group of children has provided new insights into the relevance of FC levels for diagnostic groups and outcomes. While the majority of children had abnormal FC results ranging from 51 to 249 μg/g, there were fewer than expected symptoms or investigations that were able to distinguish between diagnostic groups and FC level groups. The benefit of repeat FC testing to differentiate between diagnoses should not be overlooked as part of the longer-term diagnostic strategy.

The relevance of different FC levels is often studied in the literature as related to the accuracy of the diagnosis of IBD in children, with studied levels ranging from >50 μg/g to >800 μg/g [14]. A previous meta-analysis proposed that the FC cut-off value of 212 μg/g provided the most accurate separation between a positive or negative diagnosis of IBD in children [12]. While 17% of the current study cohort were diagnosed with IBD at the time of the index FC, this group represented less than half of the children with FC levels greater than 250 μg/g. This highlights the importance of studying additional variables assessed at the time of presentation to aid diagnosis.

The importance of considering presenting symptoms as part of the diagnostic strategy for children with raised FC is unclear as little research has been reported in this area outside the arena of IBD. When focusing on alarm symptoms specifically, the proportion of the overall cohort in the current study experiencing alarm symptoms is in line with the wider literature. Other studies have reported that up to 80% of children with both functional and organic disorders reported experiencing at least one alarm symptom [15,16], and there are mixed reports of the number of alarm symptoms experienced by children with IBD [14]. When looking for ways to differentiate between conditions, weight loss has been reported to discriminate between children with and without IBD [15], but not between children with functional and other organic GI disorders [16]. Rectal bleeding or bloody diarrhoea has been shown to distinguish between children with functional and organic GI disorders [16], and between children with and without IBD [17]. However, as in the current study, it is harder to separate out the diagnoses with high FC levels such as infectious GI and those with a new or subsequent IBD diagnosis due to commensurate rates of alarm symptoms [18]. When studied in combination, raised FC and alarm symptoms have been reported to have higher diagnostic accuracy for IBD [19]. However, it is clear from the current study and the wider literature that there is a cross-over between the presenting picture for IBD and infectious diarrhoea [20]. For children with functional GI disorders, the recommendations are to refer to a gastroenterologist if rectal bleeding persists for more than two weeks [21], and the World Health Organization defines acute diarrhoea as lasting less than fourteen days [20], thereby providing some time parameters for the consideration of presenting symptoms.

Additional serum testing carried out in conjunction with FC in the current study provided some delineation between the different FC levels, with children with FC levels 500+ μg/g having more abnormal iron studies, albumin, CRP, and platelets. This tallies with the literature whereby children with functional GI disorders have no association between FC level and abnormal serum markers for WBC, platelets, iron studies, or haemoglobin [11]. When the diagnostic groups of infectious diarrhoea, IBD diagnostic and IBD subsequent in the current research, were studied in isolation, the markers found to be indicative of a new diagnosis of IBD over the other two groups were abnormal platelets. Wider research has reported that FC levels in children with infectious diarrhoea are not associated with raised WBC or CRP [22]. When adults with infectious diarrhoea and IBD were compared, those with IBD were more likely to have abnormal platelets, albumin, WBC, haemoglobin, and ESR [23]. These abnormalities are also seen in children with raised FC at the time of IBD diagnosis [24]. Up to 50% of children with serum markers measured up to 100 days before being diagnosed with IBD may present with abnormal platelets, CRP, ESR, WBC, and haemoglobin [25]. 

With radiological testing carried out on over 30% of the current study cohort, more children in the group with FC levels 500+ μg/g had abnormal findings on abdomen/small bowel MRI and abdominal USSs than in the other groups. Sub-group analysis within the 500+ group μg/g showed that for children where the index FC was related to a new diagnosis of IBD, they were more likely to have abnormal abdominal USSs than if they had an infectious GI condition or subsequent IBD. While abnormalities may be seen in USSs in children with infectious diarrhoea [26,27,28], abdominal USSs carried out on children presenting with diarrhoea, bleeding diarrhoea, or weight loss have positive findings in approximately 20% of cases [29]. In one further study, abnormal USS findings did not distinguish between functional and organic conditions [16]. The finding in the current study cohort of more abnormal colonoscopy findings for the children with IBD is not unexpected and highlights that invasive testing such as endoscopy is required for children with a high suspicion of IBD.

For children with raised FC, the importance of timely repeat FC testing may be overlooked, as is evident in the current cohort with only 28% undergoing follow-up testing in what could be considered a meaningful timeframe. While children newly diagnosed with IBD have been shown to reduce FC to levels <250 μg/g upon the commencement of treatment within 37 weeks [30], children may present with raised FC levels greater than 100 μg/g in the years leading up to their diagnosis [31]. Conversely, FC levels in children with infectious diarrhoea reduce over time [22], as shown in the current study when this sub-group of children was retested within 180 days, showing a reduction in levels. This was also the case for functional, upper/lower GI conditions (with one exception), non-GI, and for those with an unknown outcome. 

### 4.1. Strengths 

The cohort presented in this study was comprehensively reviewed for up to 18 months following their index FC, allowing for a high level of certainty concerning the diagnostic groups for analysis. The depth of the data collected on presenting symptoms and additional testing enabled a comprehensive analysis of variables that may contribute to a high suspicion of serious organic conditions such as IBD. With no external environmental factors yet shown to influence FC levels, this current study provides a snapshot of a paediatric cohort that may be considered generalisable to the wider population [32].

### 4.2. Limitations

Primary care records were not available for review for a proportion of the study cohort managed by their primary care physician, thereby preventing the analysis of complete symptom data for all children. However, carrying out a longitudinal review of testing and referral data, which were available in tertiary care records for all participants, provides reassurance that participants were not miscategorised into diagnostic groups. In categorising functional GI disorders, no formal assessment tool such as the Rome criteria [13] was reported for use with any patient, thereby making this diagnostic category subjectively based on reported clinician opinions. This current study did not investigate the effect of age on FC levels as previous work has established widely accepted parameters applicable to the age range of children included in this work. 

## 5. Conclusions

The current study facilitated a comprehensive exploration of the diagnostic spectrum of children with raised FC. There are few tests carried out at the time of diagnosis that differentiate between certain conditions associated with very high FC levels, such as infectious diarrhoea and IBD, but when studied in conjunction, they may lead to a higher level of suspicion of serious organic disease. Repeating FC levels in children well enough to allow ‘watchful waiting’ may allow for greater distinction between conditions. Further research on the normalisation of FC levels in different diagnostic groups would add to this clinical picture. 

## Figures and Tables

**Table 1:** Diagnostic groupings for the study cohort, assigned based on immediate diagnosis and confirmed with longitudinal follow-up.

Group N (%)	Conditions, as Stated in Hospital EMR	Frequency n/N (%)
Functional 57 (18)	IBS	28/57 (49)
	Constipation	15/57 (26)
	Abdominal pain of unknown origin	9/57 (16)
	Functional gut disorder	2/57 (4)
	Faecal incontinence/Encopresis	3/57 (5)
Infectious GI 33 (11)	Viral gastroenteritis	11/33 (33)
	Viral infection confirmed (1)	
	*Rotavirus*	1/33 (3)
	Bacterial infection confirmed (17)	
	*Salmonella*	3/33 (9)
	*Yerisinia*	4/33 (12)
	*Shiga-toxin Escherichia coli*	3/33 (9)
	*Aeromonas*	2/33 (7)
	*Campylobacter*	4/33 (12)
	*Clostridium difficile*	1/33 (3)
	Parasitic infection confirmed (4)	
	*Giardia*	1/33 (3)
	*Dientamoeba fragilis*	1/33 (3)
	*Cryptosporidium*	1/33 (3)
	*Blastocystis hominis*	1/33 (3)
Upper/lower GI 48 (15)	Gluten sensitivity/coeliac disease	6/48 (13)
	*Helicobacter pylori*	5/48 (11)
	Gastritis	9/48 (19)
	GOR/oesophagitis	7/48 (15)
	Duodenal disaccharidase deficiency	2/48 (4)
	Duodenal ulcers	2/48 (4)
	Duodenal lactase high	1/48 (2)
	Gastroparesis	2/48 (4)
	Lactase deficiency	2/48 (4)
	Colonic inflammation	4/48 (8)
	Perianal abscess	1/48 (2)
	Polyposis syndrome/juvenile polyps	2/48 (4)
	Haemorrhoids	1/48 (2)
	Drug-induced enteropathy	2/48 (4)
	Hirschsprung’s	1/48 (2)
	Angiodysplasia	1/48 (2)
Non-GI 20 (6)	Mesenteric adenitis	4/20 (20)
	Gynaecological condition	7/20 (35)
	Enlarged aortic lymph nodes	1/20 (5)
	Glomerular nephritis	1/20 (5)
	Oligoarticular joint pain	1/20 (5)
	Viral illness	2/20 (10)
	Hepatic steatosis	2/20 (10)
	Splenomegaly	1/20 (5)
	Perianal contact dermatitis	1/20 (5)
Unknown 83 (27)	Primary care physician managed	64/83 (77)
	No cause found	19/83 (23)
IBD diagnostic 53 (17)	CD	38/53 (72)
	UC	15/53 (28)
IBD subsequent 16 (5)	CD	11/16 (69)
	UC	5/16 (31)

N = number/denominator, n = number/numerator, EMR = electronic medical record, IBS = irritable bowel syndrome, GI = gastrointestinal, GOR = gastroesophageal reflux, IBD = inflammatory bowel disease, CD = Crohn’s disease, UC = ulcerative colitis.

**Table 2:** Presenting symptoms for the overall cohort and categorised into groups based on location.

Symptom Location	Symptom Type	Frequency N (%)
Lower GI symptoms	Diarrhoea	126 (41)
	PR bleed	54 (17)
	Constipation	33 (11)
	Stool mucous	16 (5)
	Altered bowel habit	15 (5)
	Faecal incontinence	14 (4)
	Perianal symptoms	6 (2)
	Steatorrhea	4 (1)
	Tenesmus	1 (0.5)
General GI symptoms	Abdominal pain	201 (65)
	Bloating	23 (7)
Upper GI symptoms	Nausea	45 (15)
	Vomiting	37 (12)
	Reflux	13 (4)
	Mouth ulcers	11 (4)
	Hematemesis	4 (1)
	Gastroparesis	1 (0.5)
	Dysphagia	1 (0.5)
Extra-intestinal symptoms	Weight loss	51 (16)
	Fatigue	33 (11)
	Coryza	7 (2)
	Joint pain/swelling	6 (2)
	Rash	4 (1)
	Cough	4( 1)
	Muscle aches	4 (1)
	Urinary symptoms	4 (1)
	Fever	3 (1)

GI = gastrointestinal, PR = per rectum.

**Table 3:** Association between abnormal test results and FC level groups for those in the study cohort that had the test carried out.

Test (N)	Phi	χ^2^	*p* Value
Iron (126)	0.37	17.12	<0.001
Albumin (141)	0.46	30.05	<0.001
ESR (26)	0.48	5.86	0.053
CRP (178)	0.36	23.54	<0.001
Haemoglobin (189)	0.15	4.06	0.131
Platelets (189)	0.29	15.82	<0.001
WBC (189)	0.18	5.88	0.053
MRI abdomen/small bowel (38)	0.55	11.30	0.004
Abdominal USS (66)	0.53	18.49	<0.001
Gastroscopy (97)	0.20	4.02	0.134
Colonoscopy (112)	0.52	30.02	<0.001

Abdominal CT and abdominal X-ray were excluded from analysis due to small sample sizes. USS = ultrasound scan, MRI = magnetic resonance imaging, ESR = erythrocyte sedimentation rate, CRP = C-reactive protein, WBC = white blood count.

## Data Availability

Data are available upon reasonable request to the corresponding author A.S.D. The data are not publicly available due to being in the process of setting up a data repository.

## Supplementary Materials

The following supporting information can be downloaded at https://www.mdpi.com/article/10.3390/children11040420/s1: Figure S1. Flow chart of FC tests provided for the study, with stages of exclusion. Figure S2. Heat maps of individual participants in FC level groups (a), and diagnostic groups (b) experiencing alarm symptoms. Figure S3. The percentage of children in each diagnostic group experiencing ‘alarm’ symptoms. Figure S4. Percentage of tests carried out in overall cohort, and proportion of normal/abnormal results. Figure S5. Heat map of individual test results for all FC level groups (a) and diagnostic groups (b).
