# Supplementary material for: Exploring the Diagnostic Spectrum of Children with Raised Faecal Calprotectin Levels
Source: Children (Basel). 2024 Apr 2;11(4):420. doi: 10.3390/children11040420 (PMC11049439; doi:10.3390/children11040420)

Figure S1. Flow chart of FC tests provided for the study, with stages of exclusion.
FC = faecal calprotectin, IBD = inflammatory bowel disease, y = years, ID = identification number

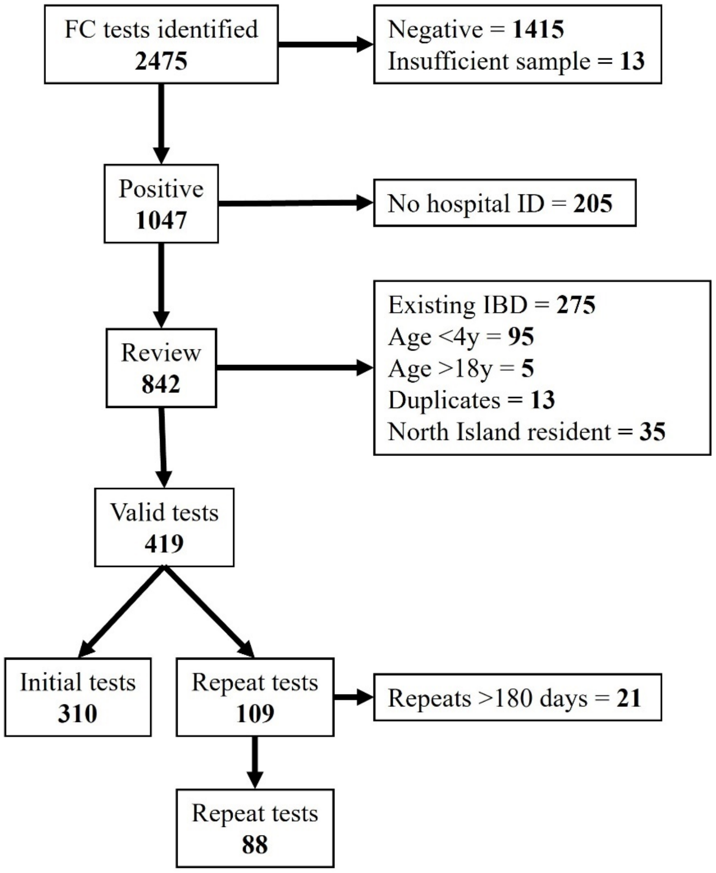

Figure S2a and S2b. Heat maps of individual participants in FC level groups (S2a), and diagnostic groups (S2b) experiencing alarm symptoms.

Figure 2a
PR = per rectum

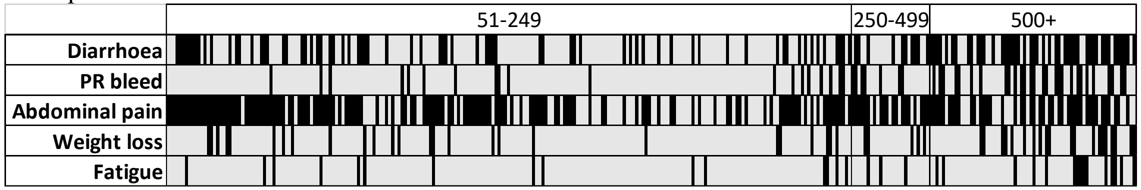

Figure 2b
GI = gastrointestinal, IBD = inflammatory bowel disease, sub = subsequent, PR = per rectum

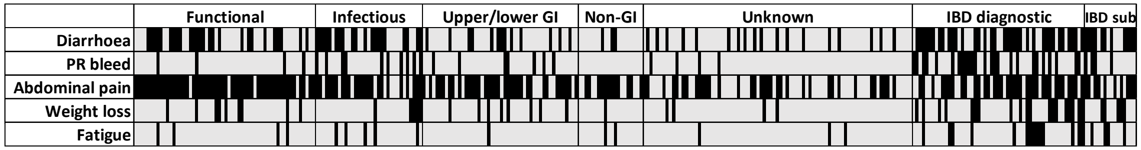

Figure S3. The percentage of children in each diagnostic group experiencing 'alarm' symptoms.

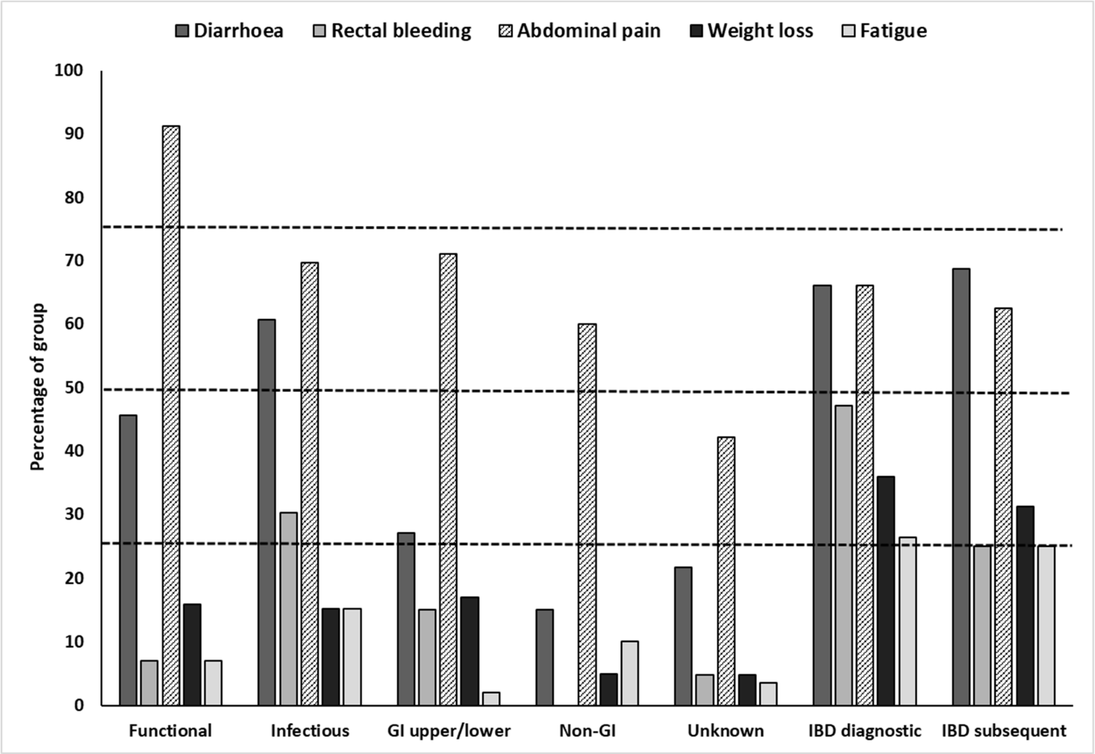

Figure S4. Percentage of tests carried out in overall cohort, and proportion of normal/abnormal results.

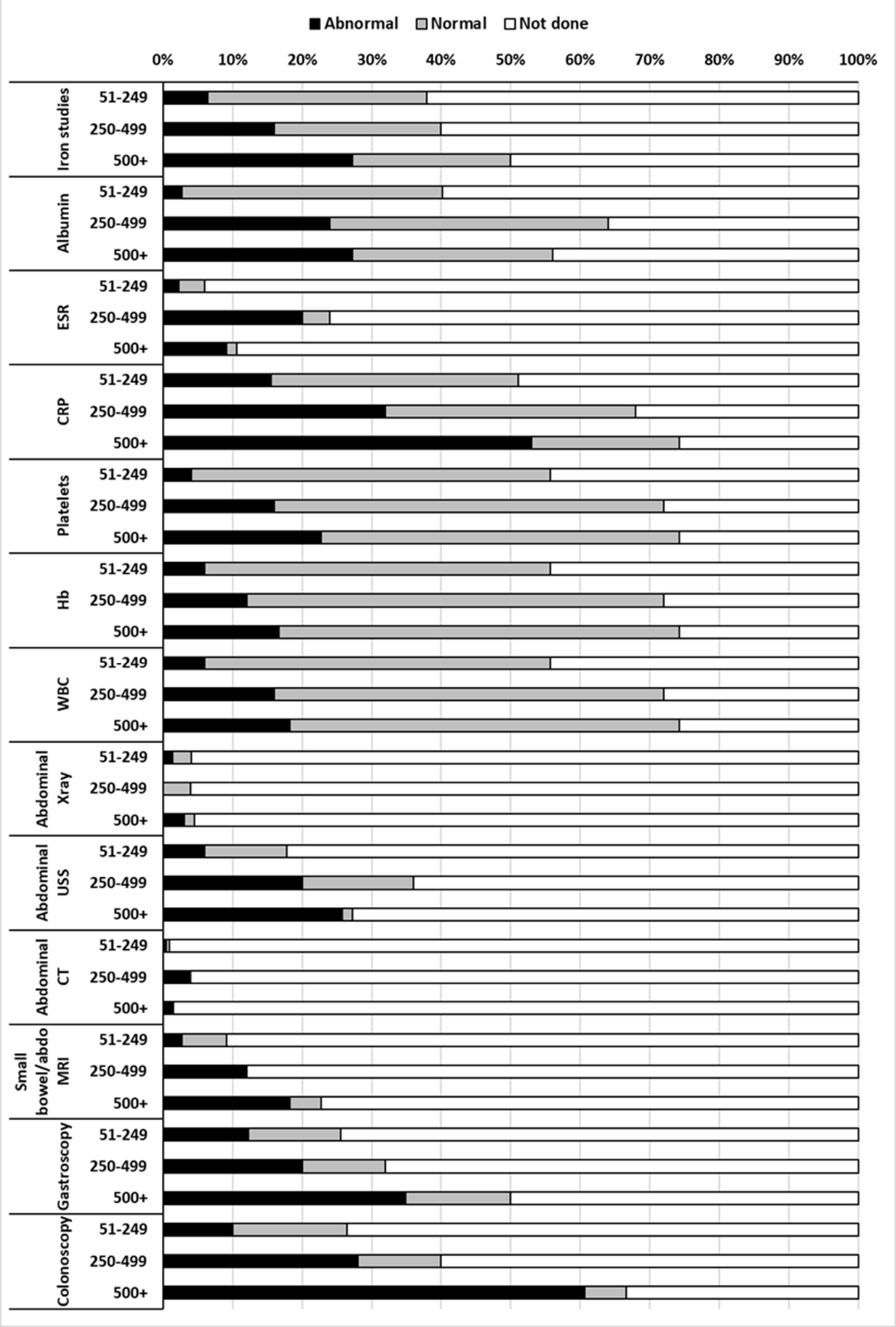

USS = ultrasound scan, CT = computed tomography, MRI = magnetic resonance imaging, abdo = abdominal, ESR = erythrocyte sedimentation rate, CRP = C-reactive protein, Hb = haemoglobin, WBC = white blood count

Figure S5a and S5b. Heat map of individual test results for all FC level groups (S5a) and diagnostic groups (S5b)

Test not done = white, test normal = grey, test abnormal = black

USS = ultrasound scan, CT = computed tomography, abdo = abdominal, ESR = erythrocyte sedimentation rate, CRP = C-reactive protein, Hb = haemoglobin, WBC = white blood count, GI = gastrointestinal, IBD = inflammatory bowel disease, sq = subsequent

Figure S5a

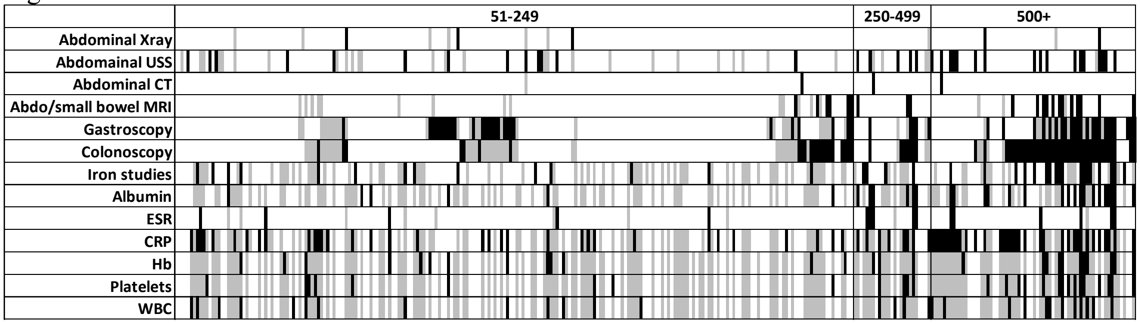

Figure S5b

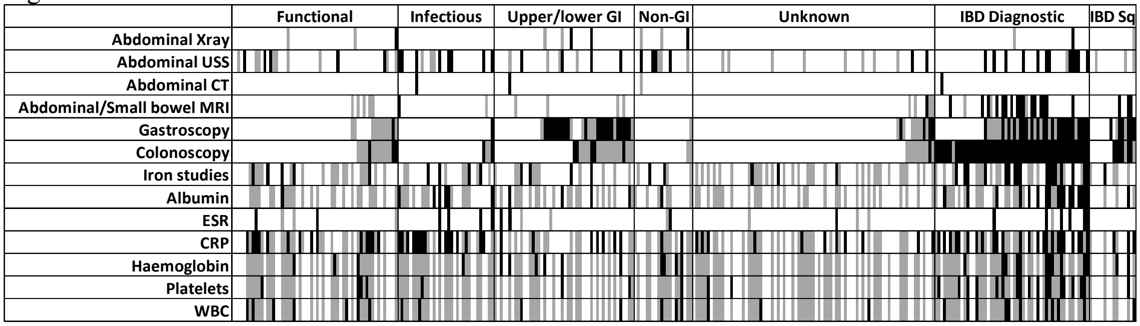

Supplement: Supplementary file 1 [file children-11-00420-s001.zip › children-2915331-supplementary.pdf]
